# Race reporting and diversity in US food and drug administration (FDA) registration trials for prostate cancer; 2006–2020

**DOI:** 10.1038/s41391-021-00361-0

**Published:** 2021-04-15

**Authors:** M. P. Lythgoe, J. Krell, P. Savage, V. Prasad

**Affiliations:** 1 Department of Surgery & Cancer, Imperial College London, Hammersmith Hospital, London, UK; 2 Department of Oncology, Brighton & Sussex University Hospital NHS Trust, Brighton, UK; 3 Department of Epidemiology & Biostatistics, University of California, San Francisco, CA USA

**Keywords:** Outcomes research, Cancer epidemiology, Cancer therapy

## Abstract

**Background:**

There is significant racial disparity in prostate cancer (PCa) in terms of incidence, treatment, and outcomes. Racial diversity and compliance with FDA race reporting guidelines in PCa drug registration trials are unknown. We analyzed racial diversity and race reporting in drug licensing trials for PCa.

**Methods:**

New drug authorizations for PCa from 2006 to 2020 were identified. The corresponding licensing trial publications were analyzed to check compliance with current FDA recommendations for race reporting. If race was unreported, the clinical trial report was analyzed to determine participant recruitment by race and lead the recruiting country.

**Results:**

During the study period, 17 new drug registrations for the management of PCa involving ten unique drugs were identified. In total, 18,455 participants were included in FDA registration trials, of which 76.3% were white or Caucasian, 7.9% Asian, 2.9% Black or African American, 0.5% American Indian or Alaskan Native, 0.1% Native Hawaiian or other Pacific Islander, 1.8% other or multiple races and 10.5% unknown. 53% of trials reported race in the licensing publication, however of this only 55% met current FDA recommendations. When the race was unreported in the licensing publication, 88% of studies had further information in the clinical study report.

**Conclusion:**

We found a significant under-representation of non-white participants in FDA drug registration trials for PCa. Race reporting in licensing publication is inconsistent and both FDA and International Committee of Medical Journal Editors guidelines are not being universally followed. Given the disproportionality of the disease burden of PCa, recruitment of Black and other minority participants to trials should be a research priority.

## Introduction

Prostate cancer (PCa) is the second most common cancer in men and shows significant disproportionally in racial prevalence [1]. In the USA, black men have a PCa incidence of 163.8 per 100,000 men, compared to 96.7 and 52.0 for white and American Indians/Alaskan natives, respectively [2]. Age-adjusted death rates are also highest in black men at 36.4 per 100,000 men, more than double that seen in white men (17.8) [2]. Causes for the racial disparity are likely multifactorial, including environmental, socioeconomic, and the underrepresentation of racial minorities in clinical trials.

Current US Food and Drug Administration (FDA) recommendations for clinical trials, introduced in 2016 recommend race reporting with a minimum of five categories; White, American Indian/Alaskan Native, Asian, Black/African American, and White Hawaiian/Pacific Islander [3]. Implementation and subsequent reporting in licensing publications of this recommendation are unknown. Furthermore, for licensing trials for which the USA is the lead participant contributor, it is unknown if these trials are representative of the PCa population. We analyzed racial representation in PCa registration trials for a period of 15 years (10 years before and 5 years after the introduction of FDA guidance) to analyze compliance with current FDA race reporting recommendations.

## Methods

A retrospective review of all new molecular entities and subsequent marketing authorizations of PCa drugs from January 2006 to July 2020 was conducted using the FDA website. Clinical trials cited on the drug licensing label for market authorization were identified using the national clinical trial identifier (NCT). The corresponding licensing trial publication was identified through clinicaltrials.gov or PubMed.

We determined whether race was reported in the corresponding licensing publication, including supplementary appendices and compliance with FDA guidance. If race was unreported or only partially reported (defined ≤3 categories), then the study report on clinicaltrials.gov or FDA website was analyzed. Additional information on participant race and recruitment by lead country was obtained to assess proportional representation based on disease population.

## Results

We identified 17 new drug registrations (with corresponding licensing publication) for the management of PCa involving ten unique drugs, including degarelix (one new license), cabazitaxel (two new licenses), denosumab (one new license), abiraterone (three new licenses), enzalutamide (four new licenses), radium-223 (one new license), apalutamide (two new licenses), darolutamide (one new license), rucaparib (one new license) and olaparib (one new license). Table 1 shows further information about licensing indication, clinical trial information, race reporting, and overall racial demographics.

**Table 1:** FDA prostate cancer licensing trials 2006–2020.

Trial name	NCT number (PMID ID of licensing study)	Drug	Prostate cancer disease subtype	No. participants	Race reported in licensing study	Race reported in the Trial report	No. Countries	Lead recruiting Country (% representation)		White/Caucasian	White Hawaiian/Pacific Islander	Black/African American	Asian	American Indian/Alaskan Native	Other/multiple	Unknown/missing
CS21	NCT00295750 (19035858)	Degarelix	Advanced	610	No	Yes	12	Unknown		511 (83.8%)	Nil	38 (6.2%)	2 (0.3%)	59 (9.7%)	Nil	Nil
TROPIC	NCT00417079 (20888992)	Cabazitaxel	Metastatic castration-resistant	755	Yes	N/A	26	USA (26.8%)		631 (83.6%)	Nil	40 (5.3%)	58 (7.6%)	Nil	26 (3.5%)	Nil
20050103	NCT00321620 (21353695)	Denosumab	Castration-resistant	1901	Limited breakdown	Yes	39	Unknown		1639 (86.2%)	2 (0.1%)	73 (3.9%)	48 (2.5%)	Nil	139 (7.3%)	Nil
COU-AA-301	NCT00638690 (21612468)	Abiraterone	Metastatic castration resistant after chemotherapy	1195	No	Yes	13	USA (41.6%)		1111 (93.0%)	Nil	43 (3.6%)	20 (1.6%)	3 (0.3%)	16 (1.3%)	2 (0.2%)
COU-AA-302	NCT00887198 (23228172)	Abiraterone	Metastatic castration resistant no previous chemotherapy	1088	No	Yes	11	USA (43.4%)		1030 (94.6%)	2 (0.2%)	28 (2.6%)	13 (1.2%)	Nil	12 (1.1%)	3 (0.3%)
AFFIRM	NCT00974311 (22894553)	Enzalutamide	Metastatic castration resistant after chemotherapy	1199	No	Yes	15	USA (24.0%)		1111 (92.6%)	1 (0.1%)	47 (3.9%)	13 (1.1%)	2 (0.2%)	Nil	25 (2.1%)
ALSYMPCA	NCT00699751 (23863050)	Radium-223	Metastatic castration-resistant (with bone metastasis)	809	Limited breakdown	No	19	UK (30.9%)		759 (93.9%)	Nil	Nil	Nil	Nil	Nil	50 (6.1%)
PREVAIL	NCT01212991 (24881730)	Enzalutamide	Metastatic castration resistant—no previous chemotherapy	1717	Yes	N/A	22	USA (14.3%)	1324 (77.1%)		2 (0.1%)	34 (2.0%)	167 (9.7%)	1 (0.1%)	Nil	189 (11.0%)
PROSELICA	NCT01308580 (28809610)	Cabazitaxel	Metastatic castration-resistant after chemotherapy	1200	Yes	N/A	22	France (10.6%)	1054 (87.8%)		Nil	25 (2.1%)	81 (6.8%)	Nil	40 (3.3%)	Nil
LATITUDE	NCT01715285 (28578607)	Abiraterone	Metastatic castrate sensitive	1199	No	No	34	Russia and Romania (15.3% each)	Nil		Nil	Nil	Nil	Nil	Nil	1199 (100%)
SPARTAN	NCT01946204 (29420164)	Apalutamide	Non-metastatic castration-resistant	1207	Limited breakdown	Yes	26	USA (27.9%)	800 (66.3%)		Nil	68 (5.6%)	140 (11.6%)	4 (0.3%)	1 (0.1%)	194 (16.1%)
PROSPER	NCT02003924 (29949494)	Enzalutamide	Non-Metastatic castration resistant	1401	No	Yes	32	USA (7.5%)	991 (70.7%)		5 (0.4%)	31 (2.2%)	230 (16.4%)	Nil	28 (2%)	116 (8.3%)
ARAMIS	NCT02200614 (30763142)	Darolutamide	Non-metastatic castration resistant	1509	No	Yes	36	Unknown	1194 (79.1%)		Nil	52 (3.5%)	193 (12.8%)	Nil	15 (1%)	55 (3.6%)
TITAN	NCT02489318 (31150574)	Apalutamide	Metastatic castration sensitive	1052	Yes	N/A	23	Unknown	719 (68.3%)		Nil	19 (1.8%)	229 (21.8%)	19 (1.8%)	47 (4.5%)	19 (1.8%)
ARCHES	NCT02677896 (31329516)	Enzalutamide	Metastatic castration sensitive	1150	Yes	N/A	24	Russia (12%)	926 (80.5%)		Nil	16 (1.4%)	155 (13.5%)	Nil	5 (0.4%)	48 (4.2%)
TRITON-2	NCT02952534 (32086346)	Rucaparib	BRCA mutant associated metastatic castration-resistant	76	Limited breakdown	No	12	Unknown	58^a^		Nil	6^a^	Nil	Nil	Nil	26^a^
PROfound	NCT02987543 (32343890)	Olaparib	Homologous recombination repair gene-mutated metastatic castration-resistant	387	No	Yes	20	Unknown	248 (64.1%)		Nil	8 (2.1%)	105 (27.1%)	Nil	3 (0.8%)	23 (5.9%)
Total	N/A	N/A	N/A	18,455^b^	N/A	N/A	N/A	N/A	14,106 (76.3%)		12 (0.1%)	528 (2.9%)	1454 (7.9%)	88 (0.5%)	332 (1.8%)	1949 (10.5%)

^a^Race reported as non-mutually exclusive; therefore, percentages have been excluded (would exceed 100%).

^b^Race subtotals exceed grand total due to TRITON-2 being reported non-mutually exclusive, percentages have been adjusted correspondingly (for total = 18,469).

The race was reported in 9 (52.9%) licensing publications. However, 4 (23.5%) provided limited information (e.g., only reporting frequency of Caucasian participants). Two of these trials had further information in the trial report and two had no further data available. For 8 (47.1%) licensing publications where no race information was reported, seven had further information within the trial report. Precise subgroup analysis by race was performed in only 2 (11.7%) studies, however, a further 9 (52.9%) studies did analyze trial participants by recruitment site continent from which some race data could be extrapolated.

Of the 18,455^1^ participants included in PCa licensing trials, 14,106 (76.3%) were white or Caucasian, 1454 (7.9%) Asian, 528 (2.9%) Black or African American, 88 (0.5%) American Indian or Alaskan Native, 12 (0.1%) Native Hawaiian or other Pacific Islander, 332 (1.8%) other or multiple races and 1949 (10.5%) unknown. Recruitment by country was reported in 11 (64.7%) out of 17 trials. The USA was the leading participant recruiter in 7 (41.1%) trials, which involved 8562 participants. Of which 6998 (81.7%) were white or Caucasian, 641 (7.5%) Asian, 291 (3.4%) Black or African American, 103^2^ (1.2%) other and 529 (6.2%) unknown.

## Discussion and conclusion

Guidelines from the International Committee of Medical Journal Editors (ICMJE) recommend, that because the relevance of race is not always known authors should, at a minimum, provide descriptive data. This study found that race reporting in FDA licensing publications does not meet the ICMJE guidance and is reported in only 53% of licensing publications^3^. This echoes the overall poor reporting rates observed in other phases 3 PCa clinical trials [4].

Following the introduction of new FDA guidance for race reporting in 2016, the reporting rate is relatively unchanged with 55% of studies meeting requirements. Reporting is also not uniform and harmonization of recording, following FDA guidance would greatly improve population analysis. Furthermore, this is consistent with a recent study by *Rencsok et al* which found 29 different race or ethnicity categories utilized in 72 PCa prevention, screening, and treatment trials from 1987 to 2016 [5].

A review by the FDA of new drug approvals between 2008 and 2013 found one-fifth of drugs demonstrate differences in exposure and/or response across racial/ethnic groups [3, 6]. Current guidance expects sponsors of clinical trials to enroll participants who reflect the demographics of the clinically relevant populations. This study concurs with the previous findings of significant under-enrollment of non-white participants in PCa trials [4, 5, 7]. Furthermore, in trials in which the USA was the leading participant recruiter, trial populations were not representative of the PCa population. Only one licensing publication acknowledged the underrepresentation of racial groups as a limitation, suggesting that racial disparity in PCa trials needs greater recognition [8]. Overall, there is a preponderance of white participants in PCa trials despite the known racial disproportionality of the disease burden. The recruitment of trial participants reflective of the burden of disease must be a research priority.

## Footnotes

1 Race reporting in TRITON-2 was not mutually exclusive, thus totals in race breakdown exceed participant total

2 Includes White Hawaiian/Pacific Islander (ten patients) and American Indian/Alaskan Native (ten patients)

3 Journals (Journal of Clinical Oncology (*n* = 2)) and Clinical Cancer Research (*n* = 1) are not present on the ICJME list of journals stating compliance with guidance (http://icmje.org/journals-following-the-icmje-recommendations/#B)
